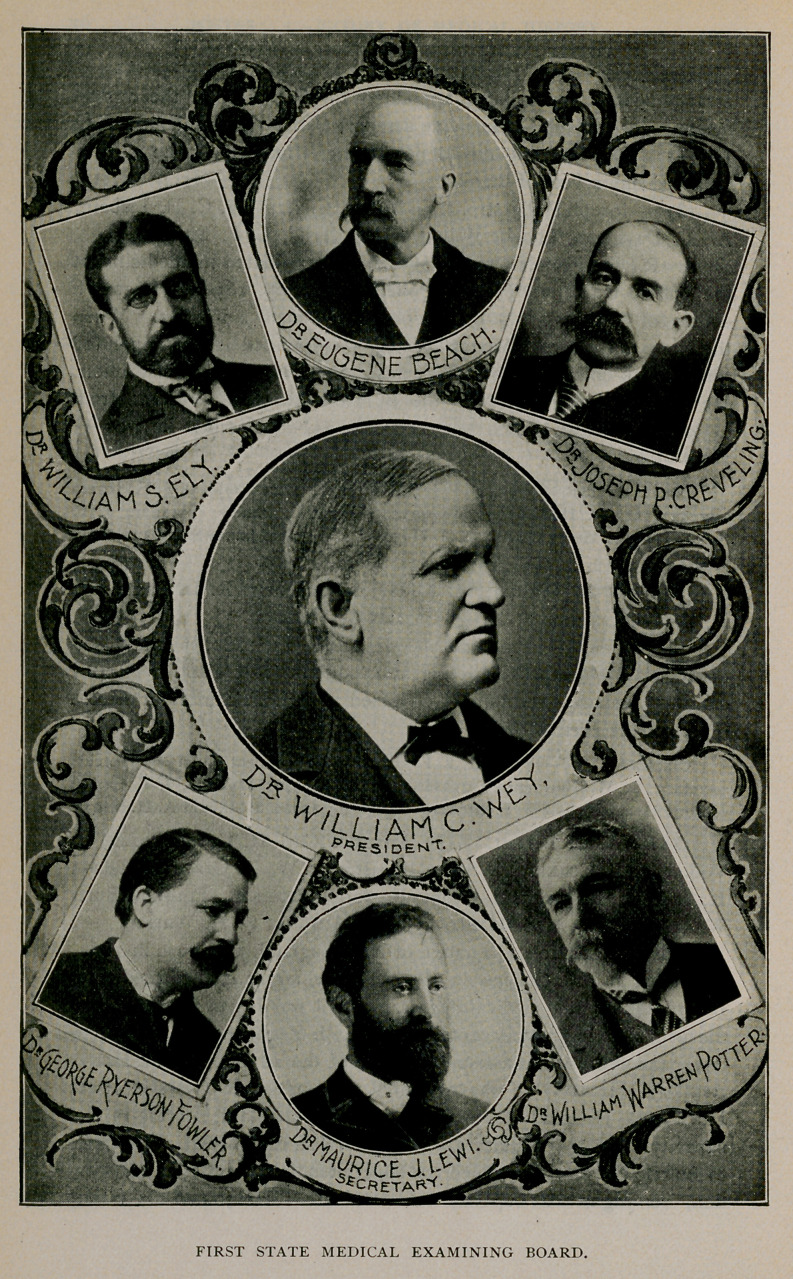# A Century of Medical History in the County of Erie.—1800–1900

**Published:** 1898-08

**Authors:** William Warren Potter

**Affiliations:** Buffalo, N. Y.


### A CENTURY OF MEDICAL HISTORY IN THE COUNTY OF ERIE.—1800–1900.

By WILLIAM WARREN POTTER, M. D., Buffalo, N. Y.

*Pioneer Physicians—Medical Societies—Medical Colleges—Hospitals—Medical Journals—Medical Officers of the Civil War—Women Physicians—History of Homeopathy—Individual Members of the Profession.*

[*Continued from the June edition.*]

1856—S. O. Almy, James B. Colegrove, Benjamin H. Lemon, William Howell, D. Devening, Edward L. Holmes, J. A. Jeyte, Jr., George Hadley and J. Condict Whitehead.

George Hadley, who joined the society in 1856, was a teacher of chemistry at the University of Buffalo from the foundation of the medical college until his death, which occurred October 16, 1877, when he was 64 years of age. He was universally loved and respected by physicians and students.

Benjamin H. Lemon was appointed demonstrator of anatomy in Buffalo Medical College in 1858, and served as such for three or four years.

Officers for 1856—President, William Van Pelt; vice-president, Frank H. Hamilton; secretary, James M. Newman; treasurer, Charles H. Wilcox; librarian, James B. Samo; primary board, Sandford Eastman, C. H. Baker and James S. Hawley; censors, P. H. Strong, C. H. Baker, R. W. Nelson, C. C. Wyckoff and C. B. Hutchins.

1857—John Gilmore, G. A. Rogers, F. F. Hoyer, Austin Flint, Jr., Sylvester Rankin, Henry Nichell, John P. Cole, Charles P. Fanner.

Austin Flint, Jr., who joined the society in 1857, was appointed professor of physiology at the Buffalo Medical College in 1858 and became editor of the BUFFALO MEDICAL JOURNAL during the same year. He was teaching physiology at the Bellevue Hospital Medical College, New York, until its union with the University Medical College in 1898.

Dr. F. F. Hoyer, of Tonawanda, is still actively engaged in the practice of medicine and was president of the society in 1880.

Dr. Henry Nichell has been a practising physician in Buffalo for more than forty years and is still so engaged.

Officers for 1857—President, Frank Hastings Hamilton; vice-president, Jabez Allen; secretary, James M. Newman; treasurer, Charles H. Wilcox; librarian, James B. Samo; primary board, Sandford Eastman, James S. Hawley, C. B. Hutchins; censors, John Boardman, P. H. Strong, Josiah Barnes, C. C. Wyckoff, G. F. Pratt.

1858—Augustus Jansen, Jesse I. Richards, J. Fletcher Stevens Wm. H. Butler, N. S. Lockwood, Charles Storck, Andrew C. Morey, Bernard Monahan.

William H. Butler was a man of sterling character, an able physician who obtained the respect of his colleagues and that of the community. He was appointed acting assistant surgeon in the army during the civil war and assigned to duty at Armory Square Hospital, Washington, D. C. He died during his service at this hospital, February 5, 1864.

Officers for 1858—President, Austin Flint; vice-president, L. P. Dayton; secretary, James M. Newman; treasurer, John Root; librarian, James B. Samo; censors, B. H. Lemon, William Gould, C. B. Hutchins, C. C. F. Gay, L. J. Ham; delegates to the state society, Charles H. Wilcox, John Boardman, P. H. Strong, William Van Pelt.

1859—J. Henry Rathbone, J. Whittaker, Charles Mead, Charles K. Winne, Samuel D. Flagg, J. R. Lothrop and William H. Mason.

Joshua R. Lothrop, who joined the society in 1859, was a man of integrity of character, possessed a high order of ability and attained conspicuous reputation as a skilful physician. He was president of the society in 1867. About this time his health began to fail. He returned to his native state, where he hoped to improve his health, but this proved futile and he died July 22, 1869, at Plymouth, Mass.

Charles K. Winne, who joined in 1859, was a son of Dr. Charles Winne. He entered the United States Army in 1861 as a medical officer and is still serving in that capacity.

Wm. H. Mason was appointed professor of physiology in Buffalo Medical College in 1860, and continued to teach in that chair until 1885, when he resigned his active labors, though he is still holding emeritus honors. His residence is Norwich, Conn.

Officers for 1859—President, L. P. Dayton; vice-president, James M. Newman; secretary, James S. Hawley; treasurer, C. C. F. Gay; librarian, James B. Samo; primary board, Sandford Eastman, John Hauenstein, Julius F. Miner; censors, B. H. Lemon, William Gould, C. B. Hutchins, William Ring, L. J. Ham.

1860—Leon F. Harvey, John Cronyn.

Leon F. Harvey served as secretary of the society from 1862 to 1866 inclusive. He was a successful practising dentist in Buffalo for many years, but always kept in touch with the medical profession. He removed to Denver, Colorado, in July, 1897.

John Cronyn came to Buffalo in 1859 from Canada and established himself at the corner of Church and Pearl streets. He rapidly gained an active professional practice and soon was appointed first as surgeon and next as physician-in-chief of the medical staff of the Buffalo Hospital Sisters of Charity, which latter office he held until his death. The medical department of Niagara University was established in 1883, largely if not principally through his instrumentality, and in that college he held the chair of principles and practice of medicine, and was president of the medical faculty from the

JOHN CRONYN, M. D.

foundation of the school until his end. In 1888, Niagara University conferred upon him the degree of Ph. D., and in 1893 that of LL. D.

Dr. Cronyn was president of the New York State Medical Association (1888), twice president of the Medical Society of the County of Erie (1875-1876), twice president of the Buffalo Medical and Surgical Association (1876-1883) and an honorary member of the Ontario Medical Association. For several years he was a member of the board of managers of the Buffalo State Hospital and a part of the time served as president of the board. He died Febiuary 11, 1898, aged 72 years.

Officers for 1860—President, William Treat; vice-president, Sandford Eastman; secretary, Samuel D. Flagg; treasurer, C. C. F. Gay; librarian, James B. Samo; primary board, Sandford Eastman, John Hauenstein, Julius F. Miner; censors, John Boardman, William Gould, C. B. Hutchins, William Ring, William H. Butler.

1861—Elias L. Bissell, Charles E. Brownell, Thomas Lothrop, P. S. Dorland.

Elias L. Bissell is still actively engaged in the practice of his profession in Buffalo and is one of the respected members of the society. He served as a medical officer during the civil war.

Thomas Lothrop, who joined in 1861, became president in 1874, and is still engaged in the daily practice of his profession. He became one of the editors of the BUFFALO MEDICAL JOURNAL in 1879 and has continued his relationship to that magazine up to the present day. He is one of the trustees of the Buffalo State Hospital, president of the Church Charity Foundation, was vice-president of the medical faculty of Niagara University and professor of obstetrics in that institution until June, 1898, when it was merged with the Buffalo University. He is honorary professor of obstetrics in the latter institution.

Officers for 1861—President, Sandford Eastman; vice-president, James B. Samo; secretary, Samuel D. Flagg; treasurer, C. C. F. Gay; librarian, C. C. Wyckoff; primary board, Edward Storck, Julius F. Miner, John Hauenstein; censors, John Boardman; William Gould, J. R. Lothrop, William Ring and H. M. Congar.

1862—Merritt H. Shaw, John McKinnon, Thomas M. Johnson.

Thomas M. Johnson, who joined in 1862, served as secretary of the society from 1866 to 1868 and was chairman of its committee of membership for many years. He was a medical officer during the war of the rebellion. He retired from the active practice of medicine about 1880 and has since been engaged in the drug business.

Officers for 1862—President, James B. Samo; vice-president, Charles Winne; secretary, Leon F. Harvey; treasurer, C. C. F. Gay; librarian, James B. Samo; primary board, C. C. Wyckoff, Edward Tobie, George Abbott; censors, John Boardman, George Abbott, J. R. Lothrop and John Cronyn; delegates to the state society, Sandford Eastman, Josiah Barnes, Horatio N. Loomis and Edward Storck.

1863—Joseph A. Peters, James S. Smith, C. W. Collier, S. W. Wetmore, Horace Tupper, William Robinson.

Of this number Dr. Smith and Dr. Wetmore are still members of the society and engaged in active practice.

Officers for 1863—President, Charles Winne; vice-president, C. C. Wyckoff; secretary, Leon F. Harvey; treasurer, William Ring; librarian, James B. Samo; primary board, C. L. Dayton, George Abbott, Edward Tobie; censors, John Boardman, John Cronyn, J. R. Lothrop, O. K. Parker and H. M. Congar.

1864—George Ayer, H. B. Horton, H. Vanguysling, E. B. Tefft, J. C. Greene, Andrew J. Houghton, J. S. Havens, O. W. Beckwith, U. C. Lynde, P. Goodyear and R. J. Curtis.

George Ayer was born at Hampton, N. H., May, 1821, and graduated from Dartmouth College in 1841. He took his medical degree in 1844, soon after which he located at Stafford, Genesee county, N. Y. He came to Buffalo in 1863 and joined the society a year later. He was engaged in active practice until within a few weeks of his death, which occurred December 8, 1877.

Joseph C. Greene was president in 1884 and is still engaged in the practice of his profession.

Officers for 1864—President, Cornelius C. Wyckoff; vice-president, George Abbott; secretary, Leon F. Harvey; librarian, James B. Samo; treasurer, William Ring; primary board, C. L. Dayton, S. W. Wetmore, Edward Tobie; censors, T. M. Johnson, M. H. Shaw, J. R. Lothrop, O. K. Parker and J. E. Peters.

1865 Jeremiah N. Brown, F. W. Bartlett, R. S. Myers, Edward Little, George W. Barr, ———[1] Gleason, John Cole, ———[1] Burgher.

Frederick W. Bartlett made application for admission to the society in 1859, action on which was indefinitely postponed. Dr. Bartlett called the matter up in a communication two years later, but the society, considering his methods of practice irregular still declined to elect him to membership. Finally, a peremptory mandamus from the Supreme Court was obtained by Dr. Bartlett, compelling the society to admit him. The society carried the matter to the court of appeals which decided in Dr. Bartlett's favor and he was admitted to membership in June, 1865. He was elected vice-president in 1894, and president in 1895. He pursued the practice of medicine until within a few months of his death, which occurred March 17, 1897.

Dr. Robert Wile, of Germany, was on motion of Dr. Hauenstein, elected corresponding member at the annual meeting in 1865 and during the session Dr. Wile demonstrated to the society the use of the laryngoscope, an instrument then coming into use.

1. Christian name does not appear on the record.

At a special meeting of the society, held February 4, 1865, Dr. William G. T. Morton gave a detailed account of his discovery of the anesthetic properties of sulphuric ether and its application in surgery, a full report of which may be found in the BUFFALO MEDICAL JOURNAL, November, 1896.

Officers for 1865—President, C. C. F. Gay; vice-president, George Abbott secretary, L. F. Harvey; treasurer, William Ring; librarian, James B. Samo primary board, Sandford Eastman, J. A. Peters, L. P. Dayton; censors, S. W. Wetmore, S. F. Mixer, J. A. Peters, J. R. Lothrop and P. H. Strong.

1866—David R. Lovejoy, F. W. Abbott, William C. Phelps, E. H. Hayen, Frank C. King, F. G. Stanley, Charles W. Bourne, Andrew Kamerling, H. S. Taft, George W. Nesbitt.

Officers for 1866—President, George Abbott; vice-president, Joshua R. Lothrop; secretary, T. M. Johnson; treasurer, William Ring; librarian, James B. Samo; primary board, L. P. Dayton, E B. Tefft and H. Vanguysling; censors, S. W. Wetmore, S. F. Mixer, J. R. Lothrop, P. H. Strong, John Hauenstein.

1867—Samuel Potter, M. E. Shaw, Henry Lapp, Conrad Diehl, B.H. Daggett, C. F. A. Nichell, G. A. Mackey and Milton G. Potter.

Henry Lapp, of Clarence, elected a member in 1867, was president in 1877, and became permanent member of the state society in 1881. He is a successful physician, in active practice at the present writing.

Conrad Diehl has been in the active practice of his profession for thirty years, during the greater part of the time one of the attending physicians at Buffalo General Hospital, was a school examiner for several years and is at present serving as mayor of the city, having been elected November 2, 1897, for the term of four years.

Milton Grosvenor Potter served as secretary of the society from 1868 until 1872, and was elected professor of anatomy at Buffalo Medical College in 1870, in which capacity he continued to teach until his death, January 28, 1878. He developed great capacity as a teacher, was a skilful physician and acquired a large practice while yet a young man. His talents were conspicuous and such as to command respect from his seniors as well as his contemporaries.

Officers for 1867—President, Joshua R. Lothrop; vice-president, John Boardman; secretary, T. M. Johnson; treasurer, William Ring; librarian, James B. Samo; primary board, H. S. Taft, W. C. Phelps, F. W. Abbott; censors, S. W. Wetmore, S. F. Mixer, Thomas Lothrop, P. H. Strong, John Hauenstein; delegate state medical society, George Abbott.

1868—Edwin R. Barnes, A. R. White, William D. Murray, ———[1] Eddy, Henry R. Hopkins, Charles B. Schuyler, David A. Chace, M. Willoughby, John Nichols, L. P. L. Parker.

Henry Reed Hopkins, who became a member of the society in 1868, has taken an active part in its proceedings since that time. Was vice-president in 1896 and president in 1897. He is professor of hygiene at Buffalo University Medical College. It was at his instance that the society formulated a medical practice act, creating a separate state medical examining board, which, with some modifications of his original draft, though retaining the fundamental idea, is the law under which all physicians who desire to practise in this state must obtain license.

At the semi-annual meeting of the society, held June 9, 1868, Dr. Gorham F. Pratt read a memoir of Dr. Cyrenius Chapin. On

1. Christian name does not appear on the records.

motion of Dr. White 1,000 copies were published at the expense of the society, 600 of which were distributed with the BUFFALO MEDICAL JOURNAL and may be found in Volume VIII., new series.

Officers for 1868—President, John Boardman; vice-president, Orlando K. Parker; secretary, Milton G. Potter; treasurer, William Ring; librarian, James B. Samo; primary board, T. M. Johnson, J. B. Samo, J. S. Smith; censors, S. W. Wetmore, S. F. Mixer, J. R. Lothrop, P. H. Strong, John Hauenstein.

1869—Hiram Taber, William H. Gail, J. W. Van Peyma, E. T. Dorland, H. B. Murray, Albert S. Rogers, William O. Taylor, W. S. Talbot, John J. Burk, Henry S. Ellwood, E. W. Williams, Loren F. Boies.

At the annual meeting held January 12, 1869, Dr. John S. Trowbridge read a memoir of his father, Dr. Josiah Trowbridge. On motion of Dr. Wyckoff it was ordered that 1,000 copies be published in pamphlet form for distribution, 600 of which were sent out with the BUFFALO MEDICAL JOURNAL. See Volume VIII., new series.

Officers for 1869—President, Orlando K. Parker; vice-president, Julius F. Miner; secretary, Milton G. Potter; treasurer, William Ring; librarian, James B. Samo; primary board, E. R. Barnes, Henry R. Hopkins, William C. Phelps; censors, Sandford Eastman, John Boardman, Milton G. Potter, E. M. Smith and J. R. Lothrop.

1870—M. B. Folwell, E. G. Harding, Julius Wenz, A. H. Crawford, Alphonse Dagenais, E. R. Lockman, James Sloan, Dyer Slocum, George W. Pattison, T. W. Parker, Robert C. Campbell and A. H. Briggs.

Albert H. Briggs, who became a member in 1870, has attained prominence in the profession of medicine and is known as a skilful practitioner of judgment. He volunteered his service in the war between the United States and Spain and was commissioned as surgeon of the 65th N. Y. Volunteers, May 1, 1898.

Mahlon B. Folwell, a native of Romulus, N. Y., who joined the society in 1870, came to Buffalo after the close of the civil war and pursued his medical studies under Dr. Wyckoff, receiving his doctorate degree from Buffalo University Medical College in 1867. He afterward became associated in practice with Dr. George N. Burwell and in December, 1882, married Florence, daughter of Leonidas Doty, of Buffalo. He was a consulting physician at Buffalo General Hospital; attending physician at the Buffalo Orphan Asylum and at the Children's Hospital; and was clinical professor of diseases of children at the medical department, University of Buffalo. Dr. Folwell was also a member of the Buffalo Academy of Medicine, Buffalo

MAHLON B. FOLWELL, M. D.

Medical Club, the Liberal, Buffalo, Saturn and University Clubs and a companion of the Military Order of the Loyal Legion.

Alphonse Dagenais, who joined in 1870, was a graduate of the Montreal School of Medicine and Surgery in 1867, and a licentiate of the Medical Society of the State of New York in 1870. He was also a member of the College of Physicians and Surgeons of Ontario, of the American Medical Association, of the Buffalo Academy of Medicine and of the Buffalo Medical Union. He attained the respect and confidence of a large community. He died March 4, 1897, aged fifty years.

Officers for 1870—President, Julius F. Miner; vice-president, William Gould; secretary, Milton G. Potter; treasurer, William Ring; librarian, James B. Samo; primary board, E. R. Barnes, Henry R. Hopkins, W. C. Phelps; censors, Sandford Eastman, John Boardman, M. G. Potter, W. O. Taylor and Henry Nichell.

1871—J. G. Bailey, Eugene H. Hickey, Rollin L. Banta, John J. Walsh, Michael F. Talbot, Dugald Macniel, John H. Wheeldon.

Dugald Macneil became a teacher of dermatology in the Medical Department of Niagara University. He died March 21, 1885.

Officers for 1871—President, William Gould; vice-president, William Ring; secretary, Milton G. Potter; treasurer, W. C. Phelps; librarian, J. B. Samo; primary board, John Boardman, O. K. Parker, F. W. Abbott; censors, M. B. Folwell, John Cronyn, C. C. E. Gay, Augustus Jansen, George Abbott.

1872—F. E. L. Brecht, W. A. Wasson, Benjamin L. Lothrop, John S. Halbert and P. W. Van Peyma.

Officers for 1872—President, William Ring; vice-president, Jabez Allen; secretary, David A. Chace; treasurer, William C. Phelps; librarian, James B. Samo; primary board, M. B. Folwell, H. R. Hopkins, M. Willoughby; censors, T. M. Johnson, C. C. F. Gay, E. R. Barnes and C. C. Wyckoff.

1873—U. C. Lynde, R. F. Hurdman, John Q. Harris, G. W. McPherson, F. A. Burghardt, G. H. Bailey, John Dambach, Joseph Fowler, Alfred T. Livingston, ——[1] Brooks.

George W. McPherson, of Lancaster, was elected vice-president in 1889 and president in 1890 and is a prominent physician in that village.

Officers for 1873—President, Jabez Allen; vice-president, Thomas Lothrop; secretary, David Chace; treasurer, William C. Phelps; librarian, James B. Samo; primary board, H. R. Hopkins, M. B. Folwell, M. Willoughby; censors, E. R. Barnes, Edward Storck, A. H. Briggs, C. C. Wyckoff and James Sloan; delegate to state medical society, William Gould.

1874—William H. Slacer, John C. Bump, L. A. Long, Edward N. Brush, W. W. Miner, Otto Thoma, Bernard Bartow, John D. Mathews, H. L. Atwood.

Edward N. Brush was for several years associate editor of the Buffalo Medical Journal and is at present superintendent of the Sheppard Asylum, a hospital for the insane at Towson, Md.

1. Christian name does not appear on the record.

Officers for 1874—President, Thomas Lothrop; vice-president, John Cronyn; secretary, David A. Chace; treasurer, William C. Phelps; librarian, James B.

ALPHONSE DAGENAIS, M. D.

Samo; primary board, H. R. Hopkins, M. B. Folwell, M. Willoughby; censors, E. R. Barnes, C. C. Wyckoff, Edward Storck, A. H. Briggs and James Sloan; delegates to the state medical society, William Gould, John Cronyn, George H. Lapham, William Ring and Joseph C. Greene.

1875—J. B. Frink, O. C. Shaw, Lucien Howe, Philip Sonneck, P. P. Bielby, John A. Pettit, C. R. Morrow, E. B. Potter, W. C. Earl, A. R. Southerland.

Officers for 1875—President, John Cronyn; vice-president, R. S. Myers; secretary, David E. Chace; treasurer, William C. Phelps; librarian, James B. Samo; primary board, H. R. Hopkins, M. B. Folwell, M. Willoughby; censors, Edward Storck, C. C. Wyckoff, A. H. Briggs, E. R. Barnes, David E. Chace.

1876—Herman Mynter, Samuel G. Dorr, S. S. Greene, J. I. Marcley, George L. Taylor, F. A. Baker.

At the annual meeting, held January 11, 1876, an exhaustive report was presented by the primary board in regard to the admission of students to the study of medicine. This report took high ground in reference to advanced medical education and attracted much attention. It was discussed by some of the most prominent members, including Drs. White, Miner and Strong. Dr. White commended it in the strongest terms.

Officers for 1876—President, John Cronyn; vice-president, Edward Storck; secretary, D. W. Harrington; treasurer, W. C. Phelps; librarian, James B. Samo; primary board, M. B. Folwell, H. R. Hopkins, P. P. Bielby.

1877—John R. McArtey, J. C. Wetzel, W. J. Packwood, W. V. Miller, H. M. Wernecke, C. O. Chester, Mary J. Moody, J. L. C. Cronyn, Louis Schade.

Mary J. Moody was the first woman admitted to membership in the society. She was also the first woman to receive the doctorate degree from Buffalo University Medical College.

Officers for 1877—President, Henry Lapp; vice-president, Edward Storck; secretary, D. W. Harrington; treasurer, J. B. Samo; primary board, M. B. Folwell, H. R. Hopkins, Thomas Lothrop and C. C. Wyckoff; censors, F. F. Hoyer, J. C. Greene, William Gould and John Cronyn.

1878—John G. Lanigan, Charles Cary, Arthur M. Barker, Francis W. Gallagher, Justin G. Thompson.

Arthur M. Barker was one of the younger physicians just rising into prominence when he died, December 6, 1887.

Officers for 1878—President, Edward Storck; vice-president, Sylvester F. Mixer; secretary, D. W. Harrington; treasurer, W. C. Phelps; librarian, J. B. Samo; censors, Henry Nichell, F. F. Hoyer, J. C. Greene; James Sloan, A. H. Briggs; delegates to the state society, Henry Lapp, H. R. Hopkins, E. N. Brush, T. M. Johnson and E. T. Dorland.

In June, 1878, the American Medical Association met in Buffalo and Dr. Thomas F. Rochester was chairman of the committee of arrangements, having been appointed to that office at the annual meeting in January. He made a report at the semi-annual meeting June 11, 1878, of the duties performed, after which the society tendered him a vote of thanks.

1879—Joseph Haberstro, J. G. Miller, C. A. Ring, C. D. Eisbein, A. R. Davidson, Phoebe Willett, H. P. Trull, E. E. Storck.

A. R. Davidson, a native of Canada, graduated in medicine at the Buffalo University Medical College, February, 1878. He gave a course of lectures at the college on materia medica in 1882. When the Niagara University Medical College was founded he was appointed professor of chemistry, toxicology and dermatology in that institution. He was managing editor of the BUFFALO MEDICAL JOURNAL from 1879 to his death, which occurred May 25, 1888, when he was 43 years of age.

Officers for 1879—President, Sylvester F. Mixer; vice-president, F. F. Hoyer; secretary, D. W. Harrington; treasurer, William C. Phelps; librarian, J. B. Samo; censors, Henry Nichell, F. F. Hoyer, J. C. Greene, James Sloan and A. H. Briggs.

1880—C. A. Wall, J. W. Keene, M. Hartwig, R. L. Banta, W. D. Bidaman, Julius F. Krug, Charles G. Stockton.

The society at its annual meeting, June 13, 1880, memorialised the legislature against restricting vaccinations, and also by a set of carefully prepared resolutions endorsed Dr. J. F. Miner for health officer of the port of New York.

Officers for 1880—President, F. F. Hoyer; vice-president, John Hauenstein; secretary, D. W. Harrington; treasurer, W. C. Phelps; librarian, J. B. Samo; censors, Henry Nichell, F. F. Hoyer, J. C. Greene, James Sloan and A. H. Briggs.

1881—W. C. Barrett, F. O. Vaughn, Carl H. Guess, Louis C. Volker, J. B. Coakley, J. Stone Armstrong, W. D. Granger, Judson B. Andrews, Benjamin H. Grove, Frederick Peterson, Franklin Burt, W. H. Jackson, A. S. Hancock, S. L. Atwater, S. H. Warren.

Judson B. Andrews, a native of New England, was born in 1834, graduated at Yale College in 1855, after which he studied medicine. Before taking his medical degree the civil war began and he joined the army, serving first as captain in the 77th Regiment, N. Y. Volunteers, and afterward as assistant surgeon of the 2d Connecticut Heavy Artillery. In 1867 he was appointed third assistant physician at the Utica State Hospital; later he became first assistant, serving in that capacity until the Buffalo State Hospital was established. He came to Buffalo in 1880, assumed the superintendency of the later institution, serving in that capacity until his death. He joined the society

FIRST STATE MEDICAL EXAMINING BOARD.

in 1881 and served as president in 1886. He was one of the most distinguished alienists of his time and inaugurated many methods that resulted in great benefit to the insane. He died at his hospital August 3, 1894, aged 60 years.

Officers for 1881—President, John Hauenstein; vice-president, T. M. Johnson; secretary, A. M. Barker; treasurer, F. W. Abbott; librarian, J. B. Samo; censors, Edward Storck, H. R. Hopkins, W. C. Phelps, A. H. Briggs, P. W. Van Peyma.

1882.—Clayton M. Daniels, Mary E. Runner, Edward Clark, E. H. Ballou, J. A. Hoffmeyer, Irving M. Snow, M. T. Kiefer, C. G. Champlain, Henry D. Ingraham, Carlton C. Frederick, Matthew D. Mann, William Warren Potter, George L. Brown, George W. York, C. A. McBeth, Walter D. Greene, Floyd S. Crego.

Officers for 1882—President, T. M. Johnson; vice-president, S. E. S. H. Nott; secretary, A. M. Barker; treasurer, F. W. Abbott; librarian, J. B. Samo; censors, Edward Storck, H. R. Hopkins, A. H. Briggs, P. W. Van Peyma and F. F. Hoyer; delegates to state society, F. F. Hoyer, S. E. S. H. Nott, A. M. Barker, H. R. Hopkins.

1883—Alvin A. Hubbell, Charles Weil, Jacob Frank, George E. Fell, Frank Hamilton Potter, Herman E. Hayd, James Wright Putnam, Willis G. Gregory, Eli H. Long, J. W. S. Hunter, John H. Pryor.

A special meeting was held April 11, 1883, to consider action on a bill to be introduced into the legislature regulating the practice of medicine. Dr. H. R. Hopkins, chairman of a special committee to consider the subject, reported at the semi-annual meeting, June 12, 1883, to recommend the passage of a bill creating a separate state board of medical examiners that should represent the several so-called systems of medical practice. The report of the committee closed with the recommendation that seven members be appointed as a committee of legislation to have full charge of this subject and to report action from time to time. The committee was composed as follows: John Hauenstein, M. D. Mann, F. S. Crego, Edward Storck, A. R. Davidson, H. R. Hopkins and A. H. Briggs.

At a special meeting held September 8, 1883, Dr. Hopkins's committee reported a bill, consisting of fifteen sections, that was acted upon seriatim, amended in important particulars and after debate was unanimously approved. This bill was subsequently introduced into the legislature through the Medical Society of the State of New York and after delays and amendments it finally became a law June 5, 1890. By this act the control of the practice of medicine, which had lapsed from the state many years before was now reclaimed, and under it no person is permitted to practise medicine in the state of New York without submitting, after graduation in a legalised medical college, to an examination by the state board of medical examiners. The authority to appoint this board was placed in the hands of the Regents of the university, and they under the nomination of the Medical Society of the State of New York appointed the following-named examiners: William Warren Potter, Buffalo; William S. Ely, Rochester; M. J. Lewi, New York; William C. Wey, Elmira; George Ryerson Fowler, Brooklyn; J. C. Creveling, Auburn; Eugene Beach, Gloversville. These names are given in the order in which they were officially announced from the Regents' office. Dr. Wey died June 30, 1897, and Dr. A. Walter Suiter, of Herkimer, was appointed to the vacancy thus created.

Frank Hamilton Potter, who became a member in 1883, was soon afterward appointed clinical assistant in surgery at the Niagara University Medical College. He went abroad for study in 1885 and afterward devoted himself to the practice of laryngology. In 1891, he was appointed clinical professor of laryngology at Buffalo University Medical College. He was a young man of promise and commanded the respect of his colleagues, companions and seniors in and out of the profession. He died July 16, 1891, aged 31 years.

Officers for 1883—President, S. E. S. H. Nott; vice-president, Henry R. Hopkins; secretary, A. M. Barker; treasurer, F. W. Abbott; librarian, J. B. Samo; censors, M. D. Mann, A. H. Briggs, P. W. Van Peyma, F. F. Hoyer.

1884—R. A. Witthaus, William Meisberger, W. A. D. Montgomery, B. G. Long, Carlton R. Jewett, C. Niemand, F. W. Sweetland, William H. Thornton, A. G. Gumaer, Mary Berkes, Herman Bauer, Roswell Park, R. M. Root, F. R. Campbell, Julius H. Potter, A. F. Helwig, W. B. Hawkins, Alpheus Prince, Herbert Mickel, Stephen Y. Howell, Louis Carmer, A. E. Persons.

Frederick R. Campbell, a native of Niagara county, took his baccalaureate degree at the University of Rochester and his doctorate degree at the University of Buffalo. He was appointed lecturer on hygiene at Niagara University Medical College in 1883 and afterward professor of materia medica and therapeutics. He was sanitary inspector for the board of health and acquired an extensive practice. He was the author of Language of Medicine, in which he displayed great erudition. He died September 14, 1888, aged 28 years.

Officers for 1884—President, J. C. Greene; vice-president, Judson B. Andrews; secretary, Edward Clark; treasurer, F. W. Abbott; librarian, J. B.

Samo; censors, Edward Storck, H. R. Hopkins, A. H. Briggs, P. W. Van Peyma, F. F. Hoyer.

1885—William Pask, James S. Porter, John Parmenter, A. B. Wilson, William G. Ring, F. P. Vandenburgh, F. W. Hinkel, C. F. Howard, Thomas G. Sheehan.

Officers for 1885—President, Judson B. Andrews; vice-president, E. T. Dorland; secretary, Edward Clark; treasurer, F. W. Abbott; librarian, J. B. Samo; censors, Edward Storck, H. R. Hopkins, A. H. Briggs, P. W. Van Peyma, F. F. Hoyer.

(*Continued next month.*)

## Figures and Tables

**Figure f1:**
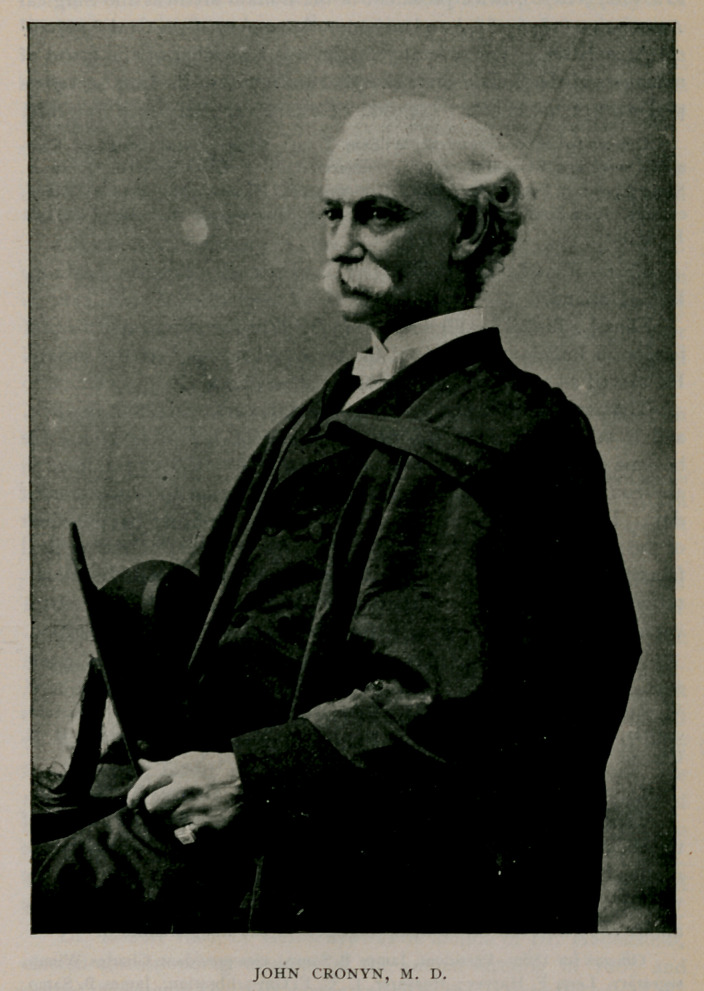


**Figure f2:**
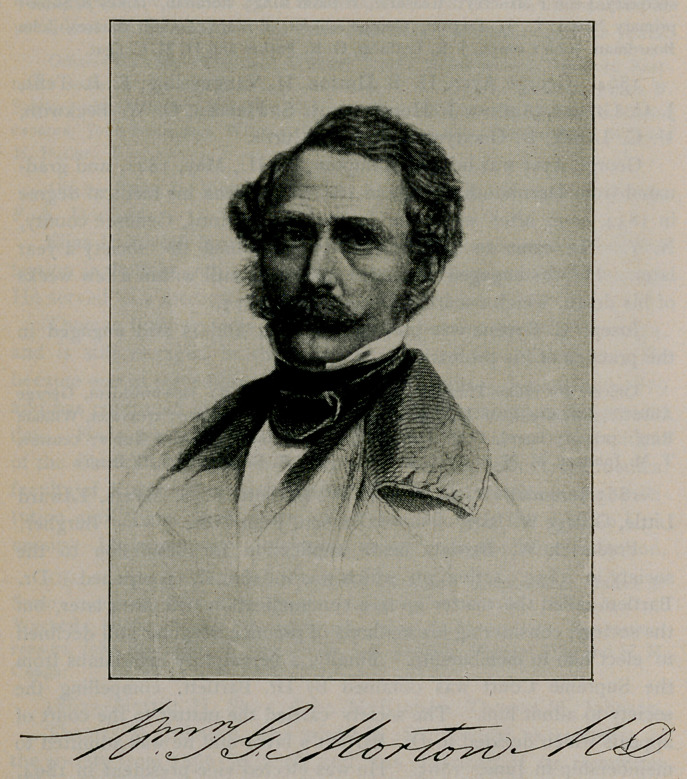


**Figure f3:**
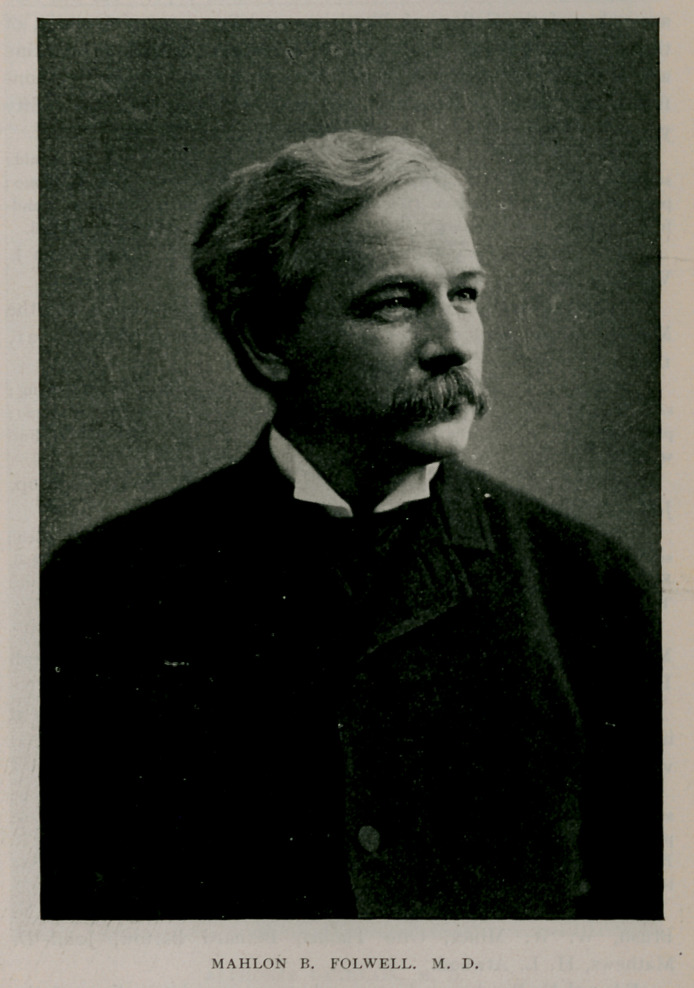


**Figure f4:**
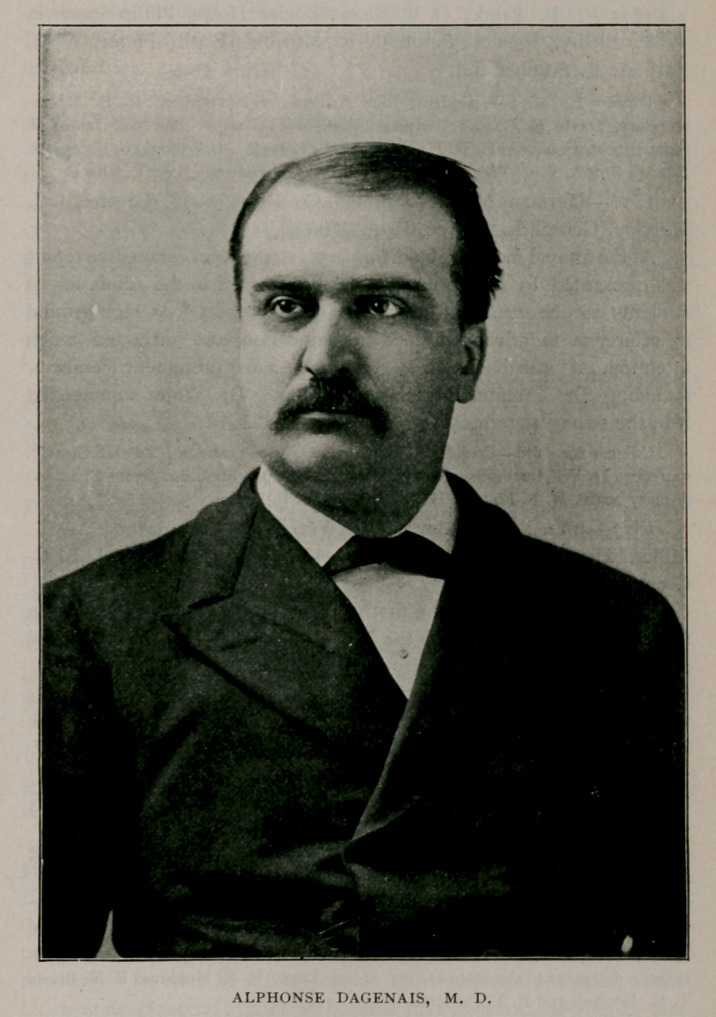


**Figure f5:**